# Hypermethylation of DDAH2 promoter contributes to the dysfunction of endothelial progenitor cells in coronary artery disease patients

**DOI:** 10.1186/1479-5876-12-170

**Published:** 2014-06-16

**Authors:** Pan-Pan Niu, Yu Cao, Ting Gong, Jin-Hui Guo, Bi-Kui Zhang, Su-Jie Jia

**Affiliations:** 1 Department of Pharmaceutics, The Third Xiangya Hospital, Central South University, Tongzipo Road #138, Changsha 410013, China; 2 Department of Cardiology, The Third Xiangya Hospital, Central South University, Changsha 410013, China; 3 Department of Pharmaceutics, The First Affiliated Hospital, Xinxiang Medical University, Xinxiang 453100, China

**Keywords:** Endothelial progenitor cells, Atherosclerosis, DNA methylation, Dimethylarginine dimethylaminohydrolase, Homocysteine

## Abstract

**Background:**

Circulating endothelial progenitor cells (EPCs) may be a biomarker for vascular function and cardiovascular risk in patients with coronary artery disease (CAD). Dimethylarginine dimethylaminohydrolase 2 (DDAH2) regulates the function of EPCs. This study aimed to examine whether hypermethylation of DDAH2 promoter contributes to impaired function of EPCs in CAD patients.

**Methods:**

Peripheral blood mono-nuclear cells from 25 CAD patients and 15 healthy volunteers were collected and differentiated into EPCs. EPCs were tested for their adhesive capability. DDAH2 mRNA expression was analyzed by real-time PCR, and the methylation of DDAH2 promoter was detected by bisulfite genomic sequencing.

**Results:**

DDAH2 promoter in EPCs from CAD patients was hypermethylated and the methylation level was negatively correlated to DDAH2 mRNA level and adhesion function of EPCs. Homocysteine impaired the adhesion function of EPCs, accompanied by lower DDAH2 expression and higher methylation level of DDAH2 promoter, compared to controls. These effects of homocysteine were reversed by pretreatment with Aza, an inhibitor of DNA methyltransferase.

**Conclusion:**

Hypermethylation in DDAH2 promoter is positively correlated to the dysfunction of EPCs in CAD patients. Homocysteine disrupts EPCs function via inducing the hypermethylation of DDAH2 promoter, suggesting a key role of epigenetic mechanism in the progression of atherosclerosis.

## Introduction

Both clinical research and animal experiments have demonstrated that endothelial repair/regeneration depends on the availability of circulating endothelial progenitor cells (EPCs), and EPCs function is disrupted by cardiovascular diseases, such as hypertension, hypercholesterolemia and coronary artery disease (CAD), as well as by other diseases such as diabetes mellitus [1-3]. Absence of sufficient circulating EPCs may in turn affect the progression of cardiovascular diseases [4]. Clinical trials conducted in CAD patients demonstrated that the level of circulating EPCs may be a surrogate biologic marker for vascular function and cumulative cardiovascular risk [4]. Furthermore, the level of circulating EPCs may predict the occurrence of cardiovascular events and death from cardiovascular causes which may help identify patients at increased cardiovascular risk [4-6].

Increased level of circulating asymmetric dimethylarginine (ADMA), an endogenous nitric oxide synthase (NOS) inhibitor, has been implicated in endothelial dysfunction in atherosclerosis [7]. Two isoforms of dimethylarginine dimethylaminohydrolase (DDAH) are responsible for the metabolization of ADMA. DDAH2 predominates in tissues that express endothelial NOS and the impairment of DDAH2 activity and/or expression rather than DDAH1 appears to be responsible for the elevation of plasma ADMA in endothelial cells of atherosclerosis [8]. Consequently, DDAH2 is recognized as a protective factor of endothelial function and a potential therapeutic target for cardiovascular diseases such as atherosclerosis, diabetes mellitus and aging [9].

Elevated homocysteine (Hcy) has been regarded as an independent risk factor for atherothrombotic vascular diseases [10]. Hcy reduces the number and function of EPCs which may be due to accelerated senescence of EPCs [11]. Our previous study has shown that hypermethylation of DDAH2 gene contributes to hcy-induced apoptosis of endothelial cells, which could be inhibited by epigallocatechin-3-gallate [12,13]. In this study we aimed to explore the possible involvement of aberrant methylation of DDAH2 promoter in the modulation of EPCs in CAD patients and determine whether the effects of Hcy on EPCs cells are mediated by the induction of DDAH2 promoter methylation.

## Materials and methods

### Study population

Twenty-five CAD positive patients (at least one coronary artery stenosis >50%) and 15 negative patients (angiographically normal) were randomly recruited from the Third Xiangya Hospital of Central South University. The characteristics of the patients were shown in Table 1. This study was approved by Ethics Committee of the Third Xiangya Hospital of Central South University, and written informed consent was obtained from all subjects.

**Table 1:** General characteristics of the patients and controls

	**Control**	**CAD patients**	***P***
	**(n = 15)**	**(n = 25)**	
Gender			0.7360
Male (%)	10 (67.70)	18 (72.00)	
Female (%)	5 (32.30)	7 (28.00)	
Age (years)	64.73 ± 3.00	67.36 ± 2.00	0.3469
BMI, kg/m2	23.10 ± 0.43	22.53 ± 0.37	0.3289
SBP, mm Hg	160.70 ± 4.00	161.00 ± 4.00	0.9438
DBP, mm Hg	91.40 ± 3.00	92.80 ± 3.00	0.8707
Creatinine, μmol/L	84.41 ± 6.17	109.6 ± 22.25	0.3943
Homocysteine, μmol/L	10.9400 ± .73	16.3400 ± 1.47	0.004
fasting blood-glucose (mmol/L)	7.06 ± 0.78	6.03 ± 0.40	0.1131
Triglyceride, ìmol/L	2.45 ± 0.48	1.97 ± 0.24	0.3320
Total cholesterol, ìmol/L	4.49 ± 0.24	4.53 ± 0.20	0.8967
LDL-C, mmol/L	2.65 ± 0.31	2.80 ± 0.43	0.8966
Positive history of diabetes (%)	5(40)	4(16)	0.2550
Positive history of smoking (%)	3(20)	9(36)	0.4770
Positive history of drinking (%)	3(20)	11(44)	0.1770

### Cell culture

Peripheral blood mononuclear cells (PBMCs) were isolated from the blood of both CAD patients and volunteers by density gradient centrifugation with Histopaque 1077 (Sigma-Aldrich, St Louis, Missouri, USA). PBMCs were resuspended in six-well plates coated with human fibronectin (Chemicon, Temecula, CA, USA), and cultured in endothelial basal medium-2 (EBM-2) (Clonetics, Walkersville, MD, USA) supplemented with endothelial growth medium-2 (EGM-2). The medium was changed every 3 days of culture.

### Flow cytometry

EPCs were cultured for 14days, then incubated with 6 μg/mL 1,1′-dioctadecyl-3,3,3′,3′-tetramethylindocarbocyanine-labeled acetylated low-density lipoprotein (acLDL-DiI) at 37°C for 2 h, and then with 10 μg/mL FITC-labeled Ulex europaeus agglutinin-1 (UEA-1) for 1 h. To detect the markers, flow cytometry was performed following routine procedures with the following primary antibodies: PE-conjugated mouse anti-CD133 antibody (1:400 dilution), PE-conjugated mouse anti-CD34 antibody (1:400 dilution), Alexa Fluor® 488-conjugated mouse anti-CD45 (1:400 dilution), and Alexa Fluor® 647 mouse monoclonal anti-VEGFR-2 antibodies (1:400 dilution). All these antibodies were from Biolegend (San Diego, CA, USA). Normal mouse, goat and rabbit IgGs were substituted for primary antibodies as negative or isotype control. After final washes in PBS, all the samples were analyzed by fluorescence-activated cell sorting (FACS) FACScalibur system (Becton Dickinson, San Diego, CA) (gating strategy for FACS was shown in Additional file 1: Figure S1), and observed in an inverted fluorescent microscope (Nikon, Japan).

### Detection of adhesion ability of EPCs

EPCs were cultured for 14 days and collected by digestion with 0.25% trypsin and centrifugation. Then the cells were resuspended in EGM-2 medium at the density of 1 × 10^5^/ml. 200 μl cell suspension was seeded in 24-well plates coated with HFN and incubated at 37°C for 2 h. Next the non-adherent cells were washed away and the adherent cells were incubated with Hoechest33342 staining solution at 37°C for 20 min, followed by washing with PBS twice. Finally, the stained nuclei were observed and counted under an inverted fluorescence microscope.

### Real-time quantitative PCR

Total RNA was isolated from EPCs using the RNeasy mini kit (Qiagen, Valencia, CA, USA). Real-time quantitative RT-PCR was performed using a Rotor-Gene 3000 (Corbett Research, Mortlake, NSW, Australia) and mRNA levels were quantified using the One Step PrimeScript RT-PCR Kit (TaKaRa, Dalian, China). The primers were as follows: DDAH2 forward 5′-GGTGCTGGGAGGTAAACTGA-3′and reverse 5′-CTAGATCTCTAGGTCATCAGGCCG-3′; GAPDH forward 5′-AACAGCCTCAAGATCATCAG-3′ and reverse 5′-GGATGATGTCTGGAGAGCC-3′.

### Detection of DNA methylation

Genomic DNA was extracted from cells with TIANamp Genomic DNA kit and the purity of the extracted DNA was judged based on A260/A280 ratio. Genomic DNA was subjected to sodium bisulfite modification by using EpiTect bisulfite kit, and nested PCR was carried out with GoTaq hot star polymerase system (Promega, WI, USA). The primers were as follows: outer pairs GTAGGGAATTTTGGAGTATTTGTTT and CTAAAAAATTAAACATCCTCTCTCC, product size: 1,013 bp, CpGs in product: 65; inner pairs GGTGGGTTAGTGATTTTGAGTTTAG and CTCCCCATACTCTCTATCTAATACAAAC, product size: 587 bp, CpGs in product: 43. PCR products were purified by gel electrophoresis, and subcloned into pGEM®-TEasy Vector System (Promega, WI, USA) for sequencing by Beijing Genomics Institute (Beijing, China).

### Statistical analysis

Data were expressed as mean ± standard deviation ($\bar{x}\pm \mathrm{SD}$). Multiple samples were compared by using one-way ANOVA, pairwise comparison was performed by LSD method. Spearman correlation coefficient was calculated by bivariate correlation analysis. All analyses were performed with SPSS 13.0 software. P < 0.05 was considered significant.

## Results

### Phenotypic characterization of EPCs

**Figure 1:**
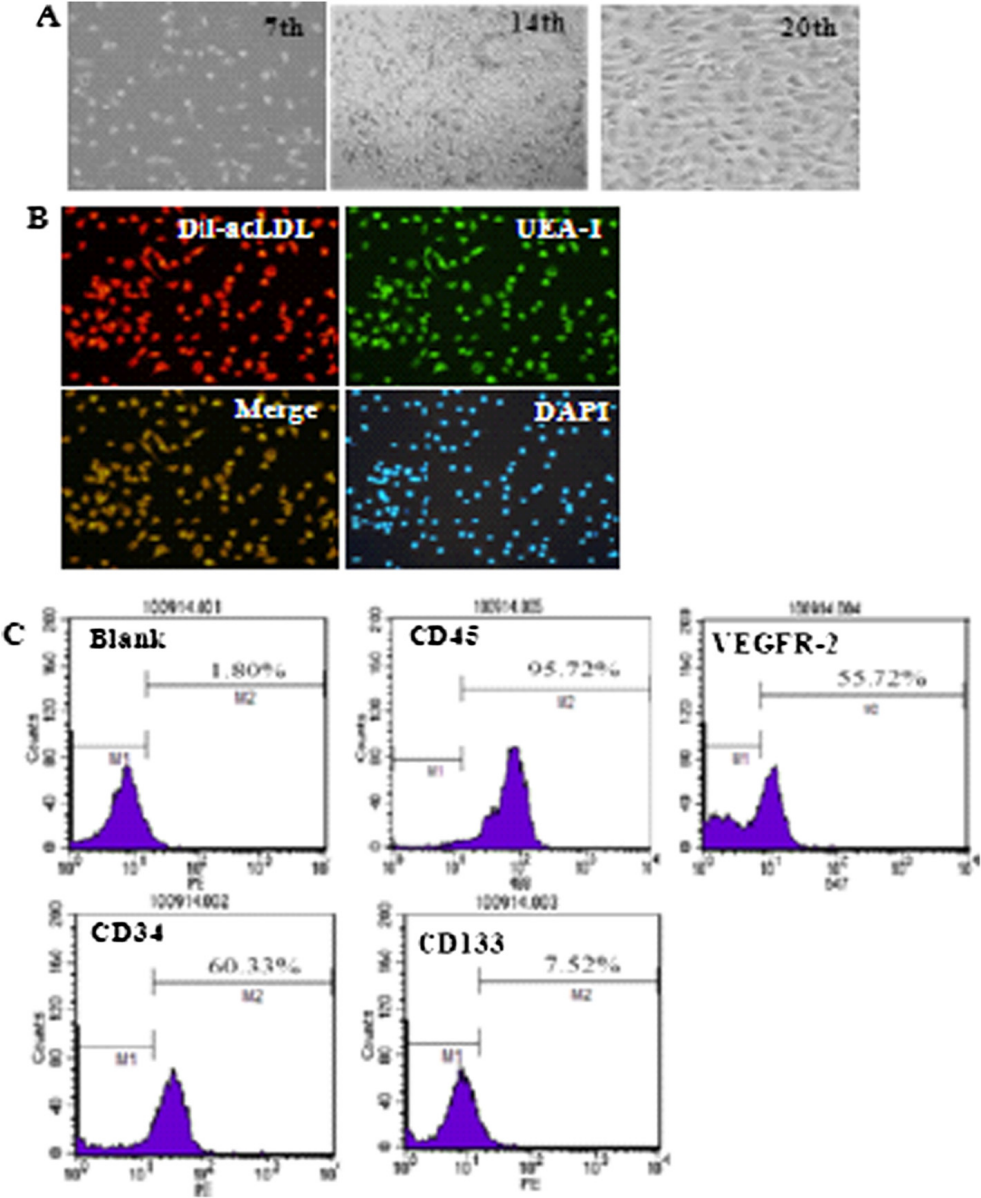
**Characterization of isolated EPCs. A**. Representative images of cultured EPCs on 7th day, 14th day, and 20th day (magnification 200×). **B**. Double-color fluorescent imaging indicated Dil-acetylated LDL incorporation and FITC lectin binding by EPCs. The nuclei was stained by DAPI (magnification 200×). **C**. FACS showed the expression of stem cell marker CD45, VEGFR-2, CD34, and CD133 in EPCs at 14th day.

EPCs were isolated from PBMCs of all the subjects. EPCs on the 7^th^ day of culture were dominantly of spindle morphology and formed colony on the 14^th^ day of culture (Figure 1A). EPCs exhibited previously defined characteristics such as acLDL incorporation and lectin binding (Figure 1B). In addition, they expressed stem cell markers CD34 (percentage: 60.3 ± 10.8%), CD45 (percentage: 95.7 ± 10.8%) and CD133 (percentage: 7.5 ± 1.6%), and endothelial cell marker VEGFR-2 (percentage 55.72 ± 14.6%) (Figure 1C).

### EPCs from CAD patients exhibit reduced adhesion ability

**Figure 2:**
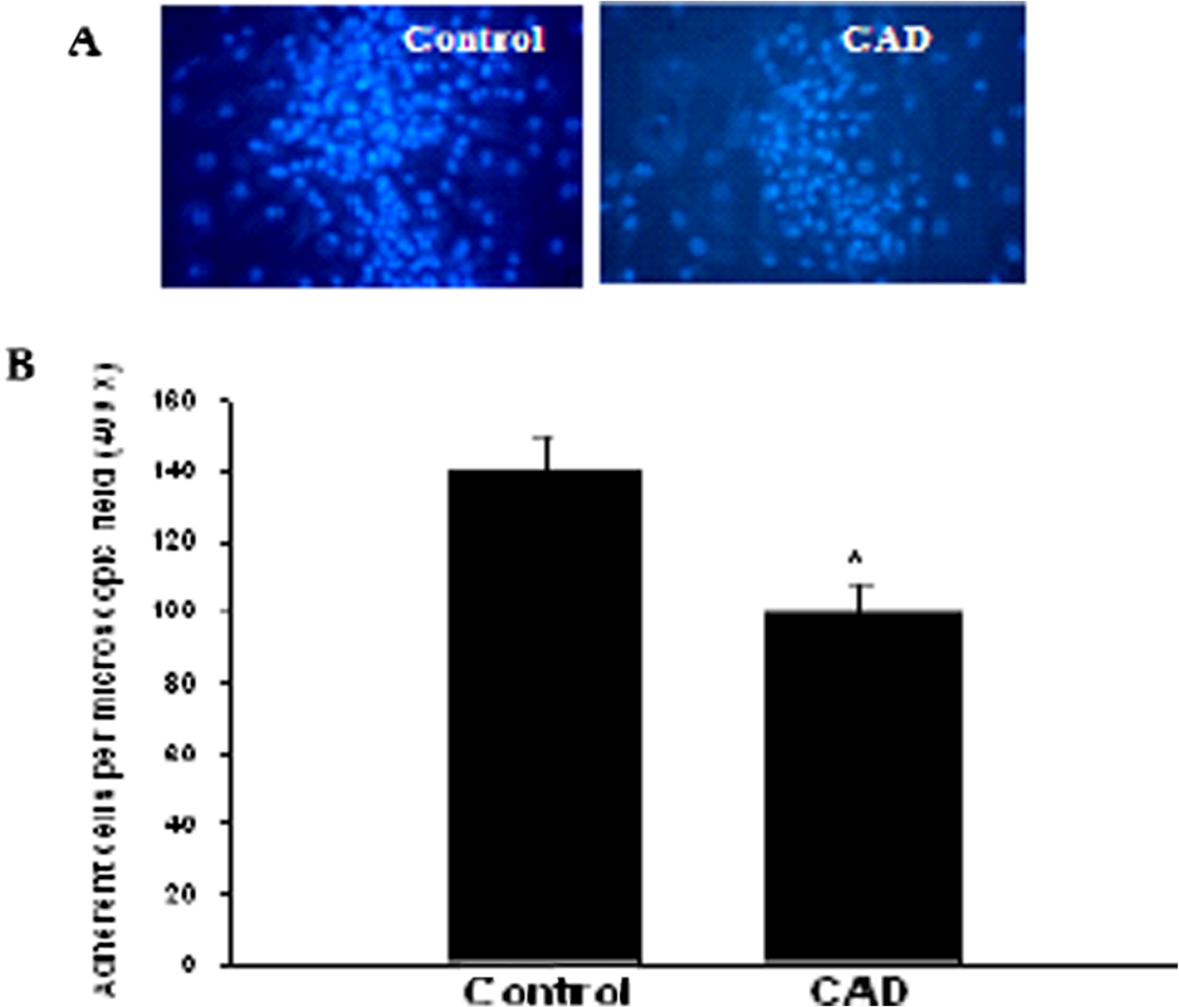
**The adhesion ability of EPCs is lower in CAD patients than in controls**. A. EPCs were isolated from CAD patients and controls and cultured for 14 days. **A**. Representative images of nuclear staining of adherent EPCs. **B**. Quantitative analysis of the number of adherent EPCs. *P < 0.05 vs. control group.

The adhesion ability of EPCs has been evaluated by the number of stained nuclei of adherent EPCs [14,15]. To examine the adhesion ability of clinically isolated EPCs, we performed Hoechest33342 staining. The results showed that the adhesion ability of EPCs from CAD patients was significantly lower than that of EPCs from control group (143.0 ± 10.8% vs. 107.2 ± 8.7%, P < 0.05) (Figure 2).

### DDAH2 gene promoter is hypermethylated in EPCs from CAD patients

**Figure 3:**
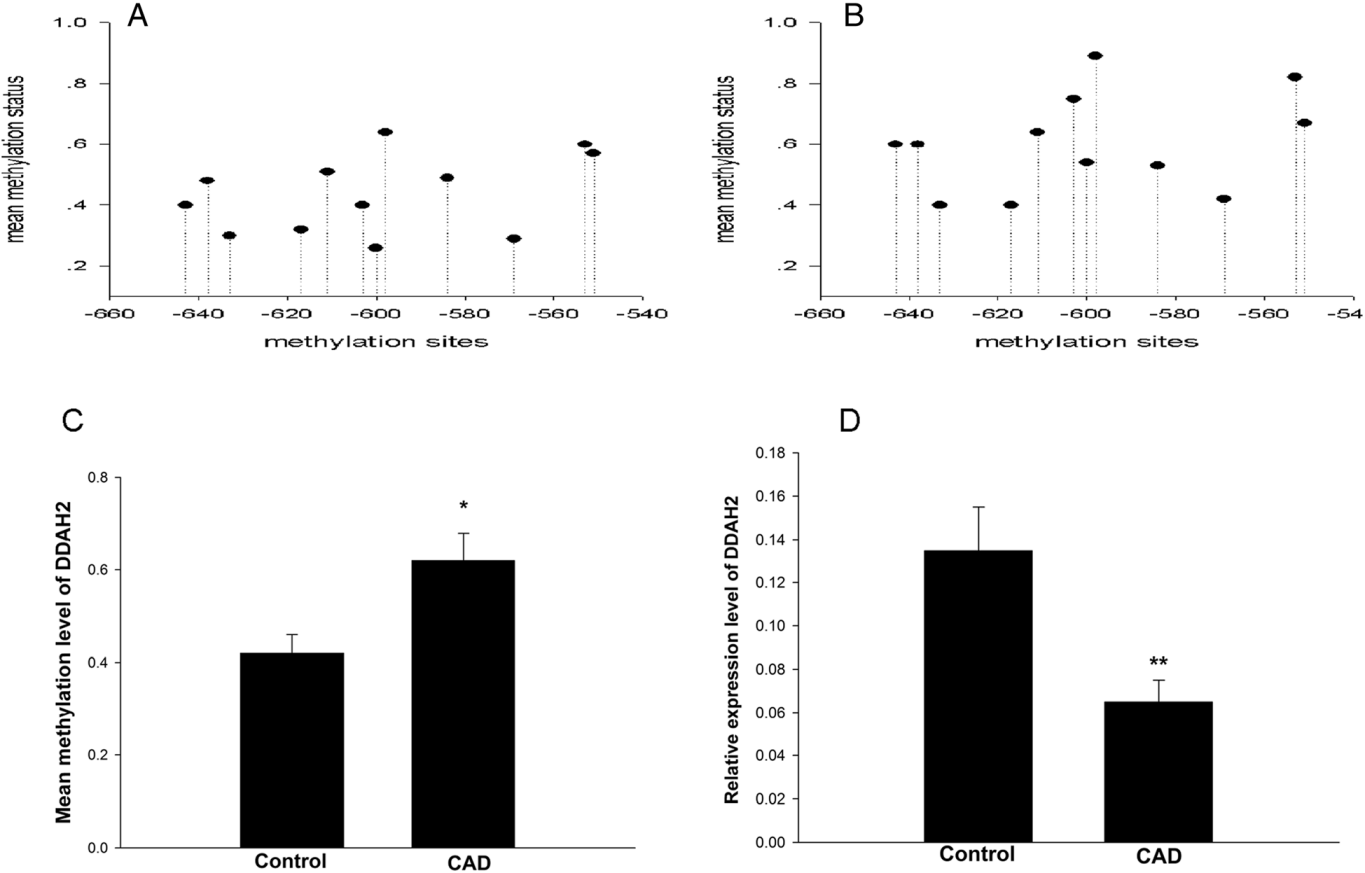
**Hypermethylation of DDAH2 promoter and reduced DDAH2 expression in EPCs from CAD patients. A**. The mean methylation status for each of the 12 CpG sites within DDAH2 promoter in EPCs from controls (n = 10). **B**. The mean methylation status for each of the 12 CpG sites within DDAH2 promoter in EPCs from CAD patients (n = 10). 1 represented fully methylated, 0 represented completely demethylated. **C**. The combined average methylation status of 7 CG pairs (positions +3144, +3162, +3170, +3200, +3229, +3261, and +3265) in DDAH2 intron 4 in EPCs was higher in CAD patients than in controls. *P < 0.05 vs. control group. **D**. The mRNA expression level of DDAH2 was lower in EPCs from CAD patients. **P < 0.01 vs. control group.

Next we detected the methylation status of CpG sites in the promoter of DDAH2 gene. The mean methylation status for each of the 12 CpG sites within DDAH2 promoter in EPCs from controls and CAD patients was shown in Figure 3A and B, respectively. We found that the average methylation status of DDAH2 promoter was significantly higher in CAD patients than in controls (P < 0.05, Figure 3C).Consistent with the hypermethylation of DDAH2 promoter in EPCs from CAD patients, real-time PCR analysis showed that DDAH2 mRNA level in EPCs from CAD patients was markedly reduced compared with controls (P < 0.01, Figure 3D).

### The adhesion ability of EPCs is negatively correlated with DDAH2 methylation

**Figure 4:**
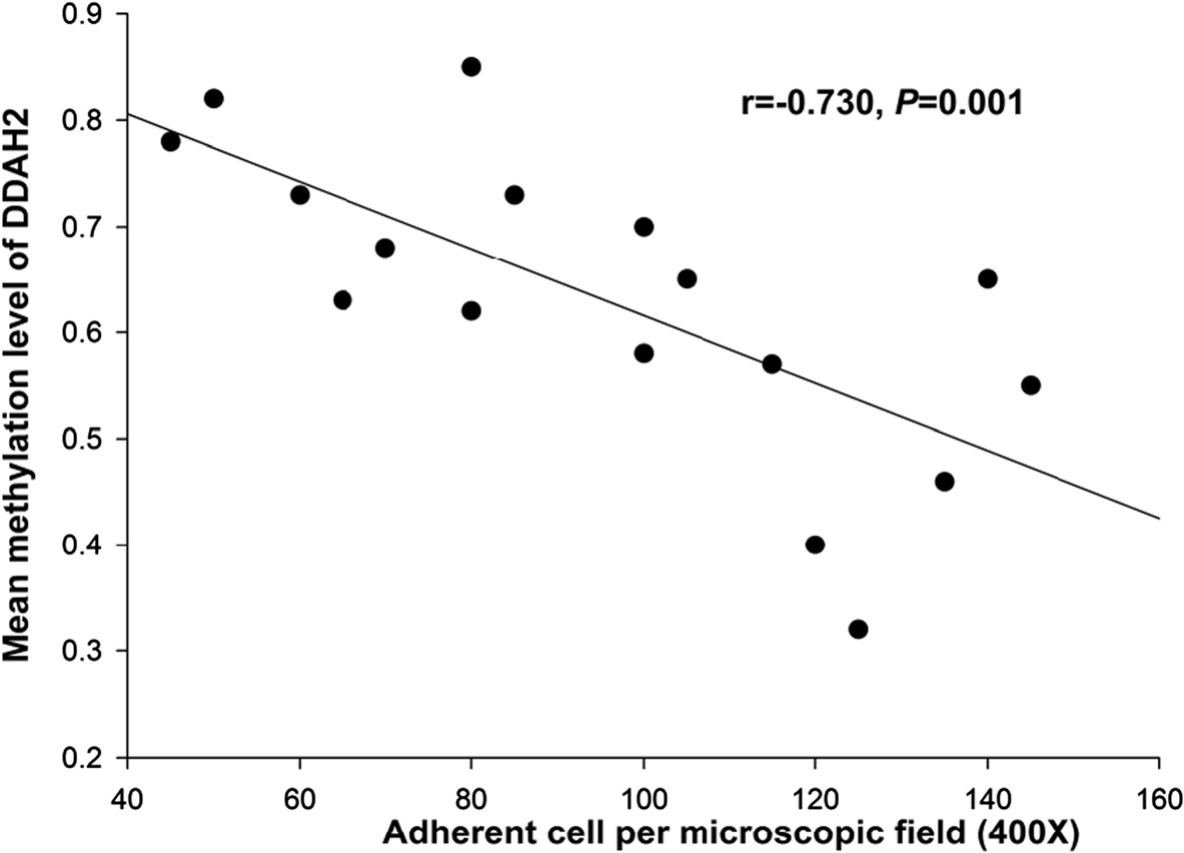
The adhesion ability of EPCs is negatively correlated with DDAH2 promoter methylation level in CAD patients (r = 0.730, P = 0.001).

Many risk factors were reported to contribute to impaired dysfunction of EPCs in CAD. Therefore, we explored whether aberrant DNA methylation of DDAH2 is involved in this process. Bivariate correlation analysis was performed for the adhesion function of EPCs and DDAH2 promoter methylation in 18 CAD patients. The results showed that the adhesion function of EPCs was negatively correlated with DDAH2 promoter methylation level in CAD patients (Pearson’s r = −0.730, P = 0.001, Figure 4).

### Hcy induces the hypermethylation of DDAH2 promoter in EPCs

**Figure 5:**
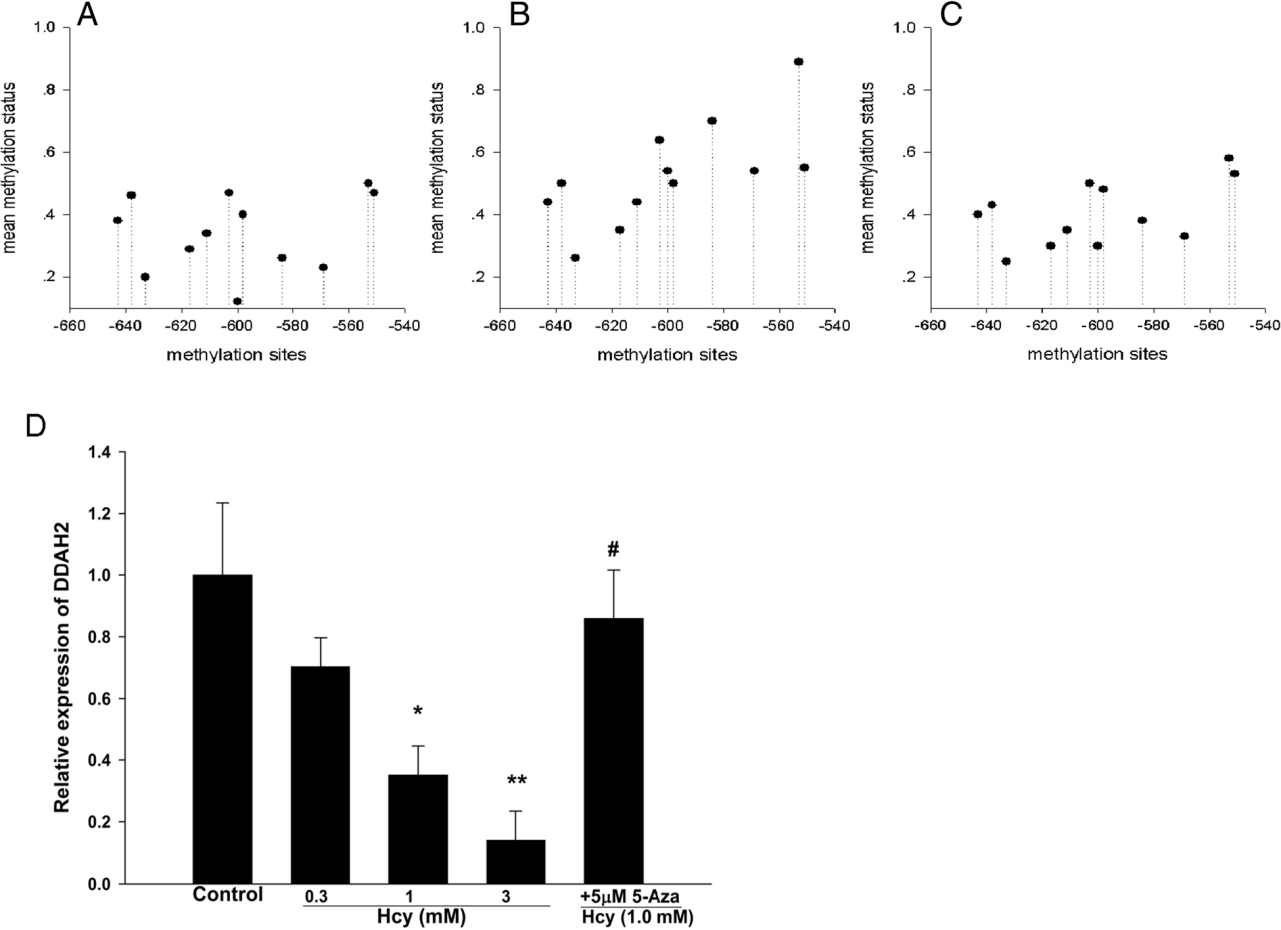
**Hcy induces the hypermethylation of DDAH2 promoter in EPCs. A**. The mean methylation status for each of the 12 CpG sites within DDAH2 promoter in EPCs untreated. **B**. The mean methylation status for each of the 12 CpG sites within DDAH2 promoter in EPCs treated with 1 mM Hcy. **C**. The mean methylation status for each of the 12 CpG sites within DDAH2 promoter in EPCs treated with 1 mM Hcy and 5 μM 5-Aza. **D**. Relative mRNA expression of DDAH2 in EPCs of different groups. Values were expressed as means ± SD (n = 3). *P < 0.05, **P < 0.01 vs. Control; #P < 0.05 vs. 1 mM Hcy treated group.

We treated EPCs from healthy volunteers with 1 mM Hcy or the combination of 1 mM Hcy and 5 μM 5-Aza (the inhibitor of DNA methyltransferase, DNMT). The mean methylation status for each of the 12 CpG sites within DDAH2 promoter in EPCs untreated, treated with 1 mM Hcy, and treated with 1 mM Hcy and 5 μM 5-Aza was shown in Figure 5A,B, and C, respectively. The results showed that the average methylation status of DDAH2 promoter was significantly higher in EPCs treated with Hcy, but 5-Aza abrogated Hcy-induced hypermethylation of DDAH2 promoter.Consistent with the effects of Hcy and 5-Aza on the rmethylation of DDAH2 promoter in EPCs, real-time PCR analysis showed that Hcy decreased mRNA level of DDAH2 in EPCs from healthy volunteers in a dose dependent manner, but 5-Aza could recover mRNA expression of DDAH2 in EPCs (P < 0.05, Figure 5D).

### Hcy decreases the adhesion ability of EPCs

**Figure 6:**
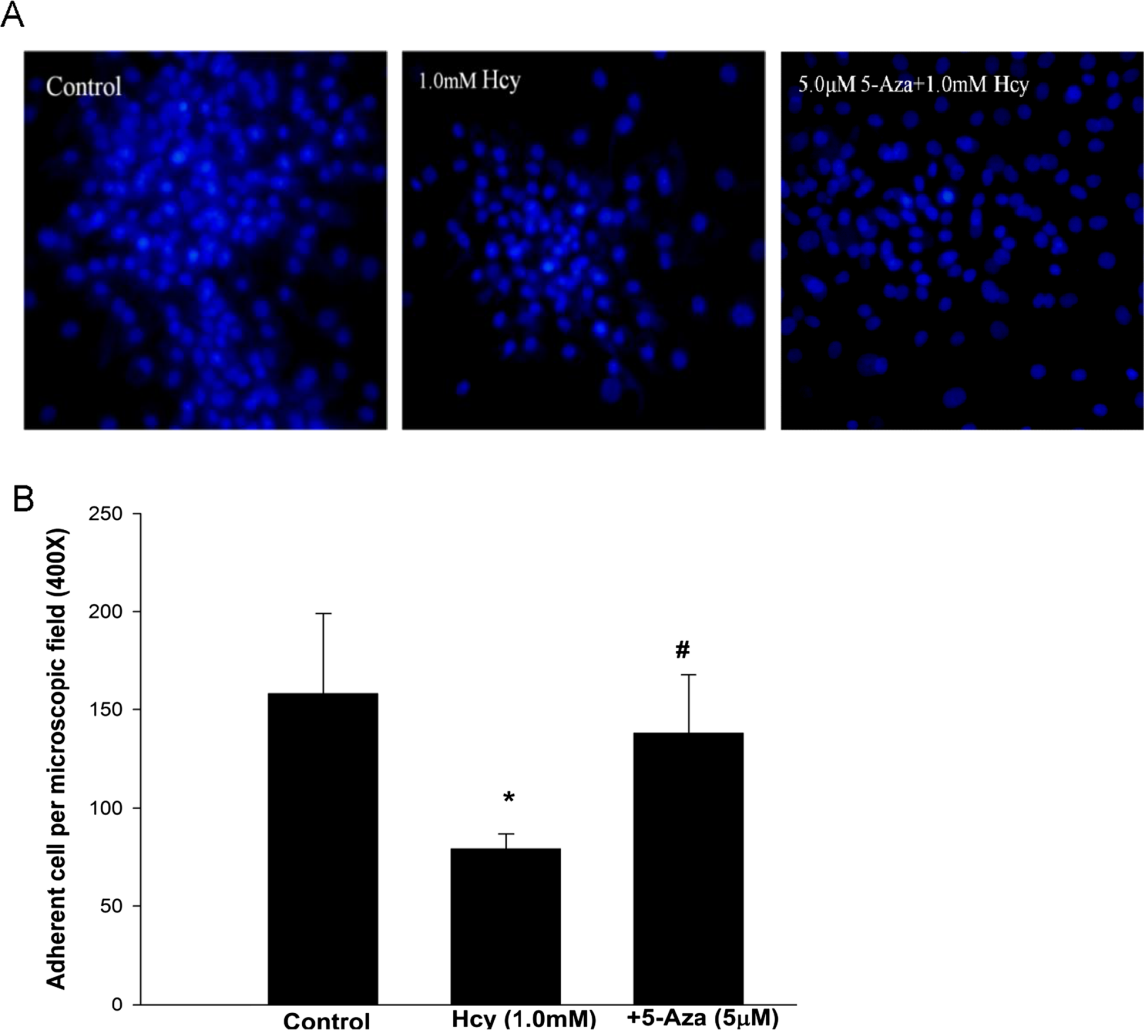
**Hcy decreases the adhesion ability of EPCs. A**. Representative images of nuclear staining of adherent EPCs in control, 1 mM Hcy treated, and 1 mM Hcy plus 5 μM 5-Aza treated groups. **B**. Quantitative analysis of the number of adherent EPCs in each group. *P < 0.05 vs. control group; #P < 0.05 vs.1 mM Hcy treated group.

Finally, to determine the biological significance of Hcy-induced hypermethylation of DDAH2 promoter, we detected the adhesion ability of EPCs untreated, treated with 1 mM Hcy and treated with 1 mM Hcy and 5 μM 5-Aza. Typical nuclear staining of EPCs was shown in Figure 6A. Quantitative analysis showed that the adhesion ability of EPCs was inhibited by Hcy, but this could be abrogated by 5-Aza (Figure 6B).

## Discussion

In the present study, we demonstrated that the adhesion function of EPCs was significantly impaired in CAD patients, accompanied by downregulated expression of DDAH2. Meanwhile, DDAH2 promoter was hypermethylated and this was negatively correlated with the adhesion function of EPCs from CAD patients. Furthermore, we isolated EPCs from healthy volunteers and treated them with Hcy. The results showed that Hcy decreased adhesion ability of EPCs, accompanied by downregulated expression of DDAH and aberrant hypermethylation of DDAH2 promoter. All these effects of Hcy were inhibited by 5-Aza, an inhibitor of DNMT. Taken together, these data suggest that aberrant hypermethylation of DDAH2 promoter may play a role in impairing the function of EPCs and contribute to CAD.

To date, no consensus has been achieved on the definition of EPCs. The CD34 + CD133 + KDR + cells are widely accepted as circulating EPCs. Flow cytometric analysis of three markers of EPCs (CD34+, CD133+ and KDR+) has been used for EPCs enumeration [16]. Therefore, in this study we chose the combination of these three markers to detect EPC populations.

One recent study showed that plaque regression was augmented by adoptive transfer of EPCs in the atherosclerosis-prone mouse model [17]. However, the bioavailability of EPCs is affected by endogenous and exogenous risk factors of atherosclerosis [18]. Exposure to ambient fine particulate matter air pollution increased the risk for cardiovascular diseases by preventing EPCs mobilization to peripheral blood [19]. Hypercholesterolemia also indirectly reduced both the availability and functionality of EPCs, thus limiting EPCs-mediated vascular repair [20]. In addition, functional impairment of EPCs results in severely reduced angiogenic capacity in vivo in metabolic syndrome [21]. Consistent with these reports, in the present study we provided clinical evidence that the adhesion ability of EPCs was significantly lower in CAD patients compared with non-CAD volunteers.

In CAD patients, it has been confirmed that ADMA is an endogenous inhibitor of the differentiation and function of EPCs [9]. DDAH2 is an endogenous catabolic enzyme of ADMA, and is expressed at relatively high level in all fetal tissues [22]. Furthermore, recent studies revealed the association between DDAH2 gene polymorphisms and CAD, type 2 diabetes and hypertension [23,24]. In our present study, we found that DDAH2 mRNA expression was downregulated in EPCs isolated from CAD patients compared with non-CAD group, suggesting that the downregulation of DDAH2 expression may contribute to impaired function of EPCs. However, the mechanism responsible for downregulated expression of DDAH2 is unclear.

DNA methylation is an important cellular mechanism that modulates gene expression associated with aging, inflammation and atherosclerotic processes. Alterations in DNA methylation profiles, including both hyper- and hypo-methylation, were present in aortas and PBMCs without detectable atherosclerotic lesion in 4-week-old mutant mice [25]. Hypermethylation of DDAH2 contributes to Hcy induced apoptosis of endothelial cells, which can be inhibited by epigallocatechin-3-gallate [12,13]. Therefore, modulating DNA methylation status of DDAH2 promoter may be a potential strategy for the treatment of endothelial dysfunction. In this study we demonstrated that DDAH2 promoter was hypermethylated in CAD patients, which may contribute to the downregulation of DDAH2 expression. Furthermore, the methylation level of DDAH2 was negatively correlated with adhesion ability of EPCs.

It has been demonstrated that Hcy level is a significant predictor of mortality, independent of traditional risk factors [26,27]. Hcy level was higher in CAD patients than in non-CAD and was inversely correlated with EPCs’ migratory capacity and ability to adhere to fibronectin [28]. In vitro, Hcy reduced the number of EPCs via the induction of apoptosis accompanied by decreased functional activity, which could be reversed by atrovastatin [29]. In this study, we found that plasma level of Hcy was higher in CAD patients than in controls, which was consistent with previous reports [30,31]. It will be interesting to elucidate the underlying mechanisms responsible for possible relationship between Hcy level and EPCs. Based on the results from CAD patients, we speculated that the effects of Hcy on EPCs may be mediated by the induction of methylation in DDAH2 promoter. We isolated EPCs from healthy volunteers and treated them with different concentrations of Hcy. We found that Hcy decreased the mRNA expression of DDAH2 in a dose dependent manner in EPCs, which can be inhibited by 5-Aza, the inhibitor of DNMT. Meanwhile, Hcy impaired the adhesion ability of EPCs and induced aberrant hypermethylation in DDAH2 promoter of EPCs. The effects of Hcy were attenuated by 5-Aza. Unfortunately, several limitations of this study should be pointed out. First, all these observations are based on cell culture system and may not reflect human condition. Therefore, further in vivo studies are needed to confirm our in vitro results. Second, our analysis of EPCs function focused only on the adhesion ability. Additional experiments to evaluate the effects of aberrant DDAH2 methylation on the migration and tube formation of EPCs will help better understand the significance of DDAH2 promoter hypermethylation.

## Conclusions

In summary, our data provide evidence that aberrant hypermethylation in DDAH2 promoter is positively related to the dysfunction of EPCs in CAD patients. Furthermore, Hcy impaired adhesion ability of EPCs and induced aberrant hypermethylation in DDAH2 promoter of EPCs, which were attenuated by 5-Aza. The present study provides new evidence for the role of aberrant DDAH2 methylation in modulating EPCs function and suggests a new epigenetic target in the treatment of atherosclerosis.

## Competing interest

The authors declare that they have no competing interest.

## Authors’ contributions

Conceived and designed the experiments: SJ. Performed the experiments: PN, YC, TG, JG. Analyzed the data: BZ. Wrote the paper: SJ. All authors read and approved the final manuscript.

## Supplementary Material

Additional file 1: Figure S1 The gating strategy for FACS analysis of the markers of EPCs. A. FITC staining. B. PE staining. Click here for file
