# Supplementary figures and images for: Hypermethylation of DDAH2 promoter contributes to the dysfunction of endothelial progenitor cells in coronary artery disease patients
Source: J Transl Med. 2014 Jun 16;12:170. doi: 10.1186/1479-5876-12-170 (PMC4069084; doi:10.1186/1479-5876-12-170)

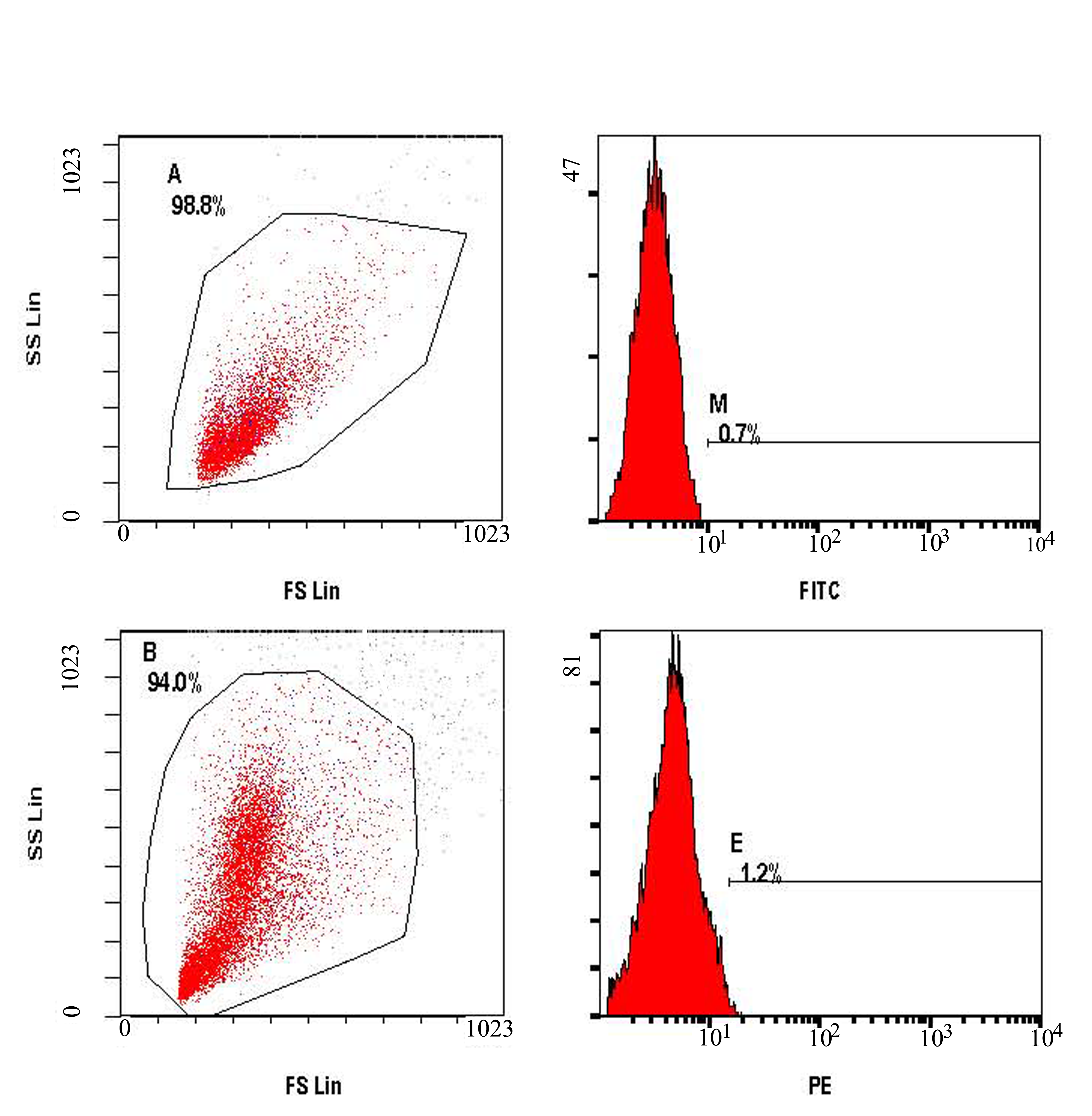

Supplement: Additional file 1: Figure S1 — The gating strategy for FACS analysis of the markers of EPCs. A. FITC staining. B. PE staining. [file 1479-5876-12-170-S1.tiff]
